# Vascular Kinin B_1_ and B_2_ Receptors Determine Endothelial Dysfunction through Neuronal Nitric Oxide Synthase

**DOI:** 10.3389/fphys.2017.00228

**Published:** 2017-04-28

**Authors:** Thássio R. R. Mesquita, Gianne P. Campos-Mota, Virgínia S. Lemos, Jader S. Cruz, Itamar C. G. de Jesus, Enilton A. Camargo, Jorge L. Pesquero, João B. Pesquero, Luciano Dos Santos A. Capettini, Sandra Lauton-Santos

**Affiliations:** ^1^Department of Physiology, Federal University of SergipeSão Cristóvão, Brazil; ^2^Department of Pharmacology, Institute of Biological Sciences, Federal University of Minas GeraisBelo Horizonte, Brazil; ^3^Department of Physiology and Biophysics, Institute of Biological Sciences, Federal University of Minas GeraisBelo Horizonte, Brazil; ^4^Department of Biochemistry and Immunology, Institute of Biological Sciences, Federal University of Minas GeraisBelo Horizonte, Brazil; ^5^Department of Biophysics, Federal University of São PauloSão Paulo, Brazil

**Keywords:** kinin receptors, nitric oxide synthases, reactive oxygen species, nitric oxide, vasorelaxation, endothelial dysfunction

## Abstract

B_1_- and B_2_-kinin receptors are G protein-coupled receptors that play an important role in the vascular function. Therefore, the present study was designed to evaluate the participation of kinin receptors in the acetylcholine (ACh)-induced vascular relaxation, focusing on the protein-protein interaction involving kinin receptors with endothelial and neuronal nitric oxide synthases (eNOS and nNOS). Vascular reactivity, nitric oxide (NO·) and reactive oxygen species (ROS) generation, co-immunoprecipitation were assessed in thoracic aorta from male wild-type (WT), B_1_- (B_1_R^−/−^), B_2_- (B_2_R^−/−^) knockout mice. Some vascular reactivity experiments were also performed in a double kinin receptors knockout mice (B_1_B_2_R^−/−^). For pharmacological studies, selective B_1_- and B_2_-kinin receptors antagonists, NOS inhibitors and superoxide dismutase (SOD) mimetic were used. First, we show that B_1_- and B_2_-kinin receptors form heteromers with nNOS and eNOS in thoracic aorta. To investigate the functionality of these protein-protein interactions, we took advantage of pharmacological tools and knockout mice. Importantly, our results show that kinin receptors regulate ACh-induced relaxation via nNOS signaling in thoracic aorta with no changes in NO· donor-induced relaxation. Interestingly, B_1_B_2_R^−/−^ presented similar level of vascular dysfunction as found in B_1_R^−/−^ or B_2_R^−/−^ mice. In accordance, aortic rings from B_1_R^−/−^ or B_2_R^−/−^ mice exhibit decreased NO· bioavailability and increased superoxide generation compared to WT mice, suggesting the involvement of excessive ROS generation in the endothelial dysfunction of B_1_R^−/−^ and B_2_R^−/−^ mice. Alongside, we show that impaired endothelial vasorelaxation induced by ACh in B_1_R^−/−^ or B_2_R^−/−^ mice was rescued by the SOD mimetic compound. Taken together, our findings show that B_1_- and B_2_-kinin receptors regulate the endothelium-dependent vasodilation of ACh through nNOS activity and indicate that molecular disturbance of short-range interaction between B_1_- and B_2_-kinin receptors with nNOS might be involved in the oxidative pathogenesis of endothelial dysfunction.

## Introduction

The kallikrein-kinin system (KKS) has multiple functions in the maintenance of cardiovascular system, including vasodilation of peripheral and coronary arteries (Madeddu et al., 1997; Heitsch, 2003), inhibition of endothelin release (Momose et al., 1993), natriuresis and diuresis (Lortie et al., 1992), inhibition of superoxide production (Madeddu et al., 2007), release of nitric oxide (NO·), and synthesis of prostaglandins and endothelium-derived hyperpolarizing factor by endothelial cells (Vanhoutte, 2004; Alhenc-Gelas et al., 2011). Altogether, these studies support the notion that KKS acts as a unique modulator of the cardiovascular system under physiological and pathophysiological conditions (Regoli et al., 2012).

Kallikrein mRNA and protein expression have been identified in blood vessels, which indicate the presence of KKS components in vascular tissue (Saed et al., 1990). KKS biologically active peptides act through two different types of G protein-coupled receptors, B_1_- and B_2_-kinin receptors (B_1_R and B_2_R), that are expressed at different molecular levels. In the vasculature, both receptors have been implicated in the activation of a wide spectrum of vasoactive pathways (Tsutsui et al., 2000; Felipe et al., 2007; Regoli et al., 2012). Indeed, B_2_R is ubiquitously expressed whereas B_1_R is virtually absent in healthy tissues, but its expression is rapidly increased under certain pathological conditions (McLean et al., 2000). However, in spite of the degree of expression, there are compelling evidence supporting that even barely expressed, B_1_R plays a physiological role and its activation induces vasoconstriction (Felipe et al., 2007) or vasorelaxation (Tsutsui et al., 2000) under unstressed conditions.

It is well-known that the endothelium-dependent vasodilatation in mouse aorta can be primarily attributed to NO· release as the result of endothelial and neuronal nitric oxide synthases (eNOS and nNOS) activity (Pohl et al., 2000; Capettini et al., 2010), which are highly regulated by changes in intracellular Ca^2+^ concentration, phosphorylation levels and, less known, by protein-protein interactions (Kone et al., 2003). Although previous studies have demonstrated molecular interactions between B_2_R/eNOS and B_2_R/nNOS (Ju et al., 1998; Golser et al., 2000), the functionality of such interactions in vascular tissue have not yet been described. Moreover, it has been shown that mice lacking B_1_- or B_2_-kinin receptors (B_1_R^−/−^ or B_2_R^−/−^) exhibit impairments in acetylcholine (ACh)-induced vasodilation (Loiola et al., 2011). Therefore, based on the above considerations, the present study was designed to evaluate the participation of kinin receptors in ACh-induced vascular relaxation, focusing on protein-protein interactions involving kinin receptors with eNOS and nNOS.

## Materials and methods

### Animals

All experimental protocols were performed in accordance with the guidelines for the humane use of laboratory animals and were previously approved by the Animal Ethics Committee of the Federal University of Minas Gerais. Male B_1_R (B_1_R^−/−^), B_2_R (B_2_R^−/−^), double kinin receptors (B_1_B_2_R^−/−^) knockout mice with their wild-type (WT) background controls (C57BL/6J, 10- to 14-wk-old, 25–30 g) were maintained in a temperature-controlled room (22–26°C) on a 12 h light/dark cycle and free access to standard mouse chow and tap water.

### Co-immunoprecipitation and western blot

Western blots were performed as previously described (Macedo et al., 2016). Thoracic aortas were homogenized in ice-cold lysis buffer (in mM: 150 NaCl, 50 Tris-HCl, 5 EDTA.2 Na and 1 MgCl_2_, pH 8.0; 1% Triton X-100, 1% NP-40, 1% sodium deoxycholate, 0.1% sodium dodecyl sulfate enriched with a protease inhibitor cocktail; Sigma FAST, Sigma, St. Louis, MO). Homogenates were cleared by centrifugation at 13,000 × g for 15 min at 4°C and protein content was quantified by Lowry assay. To detect protein complexes, proteins of interest were immunoprecipitated by incubating precleared lysates (100–500 μg protein per sample) with anti-B_1_R (5 μg; goat polyclonal, #sc-15048), anti-B_2_R (5 μg; rabbit polyclonal, #sc-25671), anti-eNOS (1 μg; rabbit polyclonal, #sc-654), or anti-eNOS (1 μg; rabbit polyclonal, #sc-8309) antibodies (all from Santa Cruz Biotechnology, USA). For kinin receptors immunoprecipitation, sample-antibody (500 μg:5 μg ratio) binding complex was carried out overnight at 4°C with constant end-over-end mixing, followed by incubation with 50 μL of Protein G Magnetic Beads (Dynabeads®, Invitrogen, #10003D) for 3 h at 4°C. For NOS immunoprecipitation, sample-antibody (100 μg:1 μg ratio) binding complex was carried out for 2 h at 4°C, followed by incubation with 10 μL of protein A/G-Sepharose beads (ThermoScientific, Waltham, USA, #53132) at 4°C overnight. As IgG control, protein extracts (500 μg) were also immunoprecipitated with normal rabbit serum (Santa Cruz Biotechnology, USA, #sc-2027) in order to assess the specificity of our antibodies. Afterwards, beads were washed 3x with lysis buffer and pelleted to remove unbound proteins. Precipitated proteins were eluted from the beads by boiling in Laemmli buffer, resolved using 10% gel electrophoresis (SDS-PAGE) and transferred onto a nitrocellulose membrane (Merck-Millipore, USA). For WB, membranes were incubated with anti-eNOS (1:1000, mouse monoclonal, #N38620, Transduction Laboratories), anti-nNOS (1:1000, mouse monoclonal, #N31020, Transduction Laboratories), anti-B_1_R (1:1000, #sc-15048, Santa Cruz Biotechnology, USA) and anti-B_2_R (1:1000, #sc-25671, Santa Cruz Biotechnology, USA) at 4°C overnight, followed by appropriate horseradish peroxidase-conjugated secondary antibodies (dilution 1:10000; Sigma, USA). Immunodetection was performed using enhanced chemiluminescence (Luminata strong™—Western HRP substrate, Merck-Millipore, USA) and images acquired using radiographic film or ChemiDoc XRS system (BioRad, USA).

### Vascular reactivity

Rings from the thoracic aorta were obtained and processed as previously described (Capettini et al., 2008). Vessels were transferred to an organ bath containing Krebs-Henseleit (K-H) solution (in mM: 110.8 NaCl, 5.9 KCl, 25.0 NaHCO_3_, 1.07 MgSO_4_, 2.49 CaCl_2_, 2.33 NaH_2_PO_4_, and 11.51 glucose, pH 7.4) maintained at 37°C and then, resting tension was adjusted stepwise to reach 0.5 g and allowed to stabilize for 1 h. ACh or substance P was added in increasing cumulative concentrations once the response to phenylephrine (0.1 μM) had stabilized. Preparations were then incubated for 20 min in the presence or absence of a B_1_R-selective antagonist (Des-Arg^10^ HOE 140; 1 μM), or a B_2_R-selective antagonist (HOE 140; 1 μM; Wirth et al., 1992; Regoli et al., 1998), a non-selective NOS isoforms inhibitor (N^G^-nitro-L-arginine, L-NNA; 1 μM; Pérez et al., 2009), a selective nNOS inhibitor [1-(2-trifluoromethylphehyl) imidazole, TRIM; 100 μM; Handy and Moore, 1997] or a superoxide dismutase (SOD) mimetic (Mn (III) tetrakis [1-methyl-4-pyridyl] porphyrin, MnTMPyP; 10 μM; Fontana et al., 1999). After pre-incubation period, a second cumulative concentration-response curve for ACh was constructed. For endothelium-independent vasorelaxation, the endothelium was removed by gently rubbing the lumen of the aortic rings. The absence of endothelium was confirmed by the lack of relaxant response to ACh in aortic rings pre-contracted with phenylephrine. Subsequently, concentration–response curve for sodium nitroprusside (SNP) was constructed. Mechanical activity was recorded isometrically using a force transducer (World Precision Instruments, Inc., Sarasota, FL, USA) connected to an amplifier-recorder (TBM-4 model; Word Precision Instruments, Inc., Sarasota, FL, USA) and a personal computer equipped with an analog-digital converter board (AD16JR; Word Precision Instruments, Inc., Sarasota, FL, USA), using WinDaq Data Acquisition software (Dataq® Instruments, USA).

### Measurements of NO· production and superoxide generation

NO· production in the aorta was determined using a fluorescent cell-permeable dye, 4,-amino-5 methylamino-2′,7′-diaminofluorescein diacetate (DAF-FM, 10 μM; Molecular Probes) as previously described (Mota et al., 2015). Intracellular superoxide levels were assessed using dihydroethidium (DHE, 10 μM; Calbiochem). Freshly isolated aortas were incubated with dyes and loaded for 30 min at 37°C in K-H solution and then washed for 30 min. Thoracic aorta segments were frozen and cut into sections that were 10 μm thick. For some experiments, fresh aortic rings from WT mice were incubated in the presence or absence of Des-Arg^10^ HOE 140, HOE 140 (1 μM) or MnTMPyP (10 μM) for 30 min prior to ACh stimulation (10 μM, 30 min). Images were recorded using a fluorescence microscope (IX2-ICB, Olympus®, USA), and image analyses were performed in ImageJ software (NIH).

### Statistical analysis

All data are expressed as mean ± SEM. Vascular responses are expressed as % relaxation of the tone induced by phenylephrine. Significant differences between groups were determined using Two-way ANOVA followed by Bonferroni's *post-hoc* tests to compare the concentration-response curves obtained in aortic rings. Fluorescence microscopy images were analyzed according to the intensity of the fluorescence per area, both represented in arbitrary units (a.u.). The delta of the area under the curve was calculated as the difference between the concentration-response curves in the presence and the absence of MnTMPyP. One-way ANOVA followed by Bonferroni's *post-hoc* tests were used for all other analyses. All statistical comparisons were made using GraphPad Prism 5 (GraphPad Software Inc., San Diego, CA, USA) and values of *P* < 0.05 were considered to be statistically significant.

## Results

### Protein-protein interactions between constitutive NOS isoforms and kinin receptors

**Figure 1:**
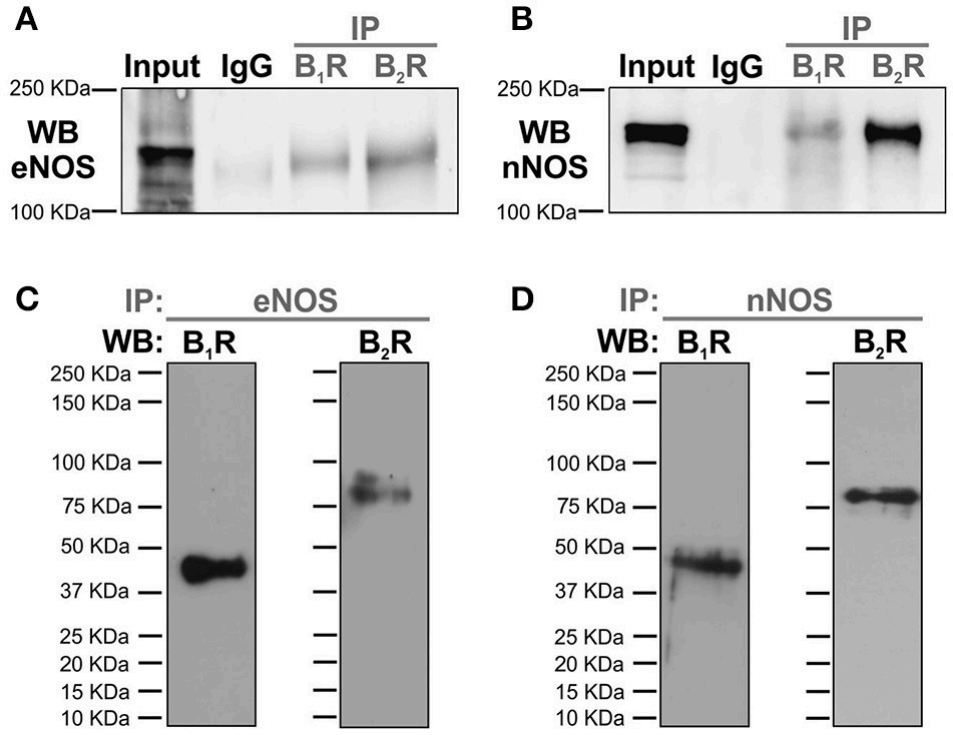
**Protein-protein interactions between constitutive NOS and kinin receptors**. Thoracic aorta proteins of wild type mice were used for immunoprecipitation experiments (IP). **(A,B)** Non-precipitated aortic lysates was used as a positive control (input, 50 μg of protein), whereas immunoprecipitation with normal rabbit serum was used as an IgG control. Proteins were immunoprecipitated using anti-B_1_R or anti-B_2_R antibody followed by WB with anti-eNOS **(A)** or anti-nNOS **(B)**. **(C,D)** Proteins were immunoprecipitated using anti-eNOS or anti-nNOS antibody followed by WB with anti-B_1_R **(C)** or anti-B_2_R **(D)**. Data shown are representative of four separate experiments, each of which provided nearly identical results.

In order to identify the existence of protein-protein interactions involving kinin receptors and constitutive NOS in native vascular tissue, thoracic aortas from WT mice were lysed and proteins were immunoprecipitated with anti-B_1_R, anti-B_2_R, anti-eNOS, and anti-nNOS antibodies. As shown in Figures 1A,B, the positive control, non-precipitated aortic lysate (input), show a strong signal at proper molecular weight, whereas IgG signal was barely detected (Figure 1A) or absent (Figure 1B) in samples immunoprecipitated with normal rabbit serum. Moreover, we show that eNOS (Figure 1A) and nNOS (Figure 1B) physically interact with B_1_- and B_2_-kinin receptors. We further validate our findings by performing opposite protein immunoprecipitation experiments (Figures 1C,D).

### Vascular reactivity

**Figure 2:**
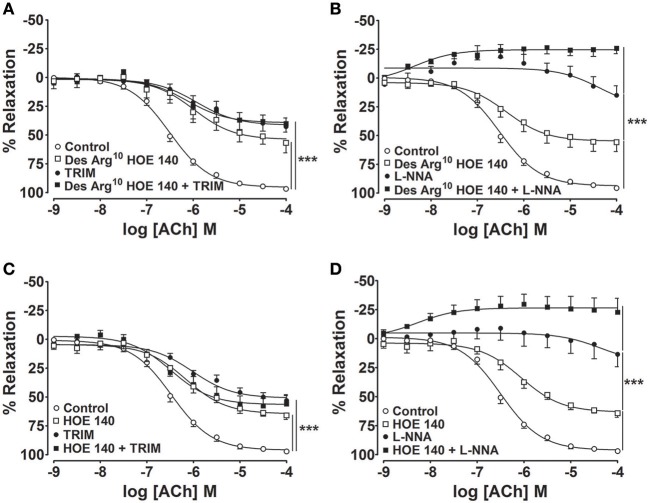
**Pharmacologic antagonism of B_**1**_- or B_**2**_-kinin receptors attenuates ACh-induced vasodilation**. Cumulative concentration-response curves for ACh were determined using an antagonist of the B_1_-kinin receptor **(A,B**; Des-Arg^10^ HOE 140; 1 μM) or B_2_-kinin receptor **(C,D**; HOE 140; 1 μM) in combination with a selective inhibitor of nNOS **(A,C**; TRIM; 100 μM) or constitutive NOS (**B,D**; L-NNA; 1 μM). The results are expressed as mean ± SEM for 8–10 experiments in each group. ^***^P < 0.001.

Based on our findings that both B_1_- and B_2_-kinin receptors are expressed and physically interact with nNOS and eNOS, we next sought to investigate the functionality of these interactions. To address this question, we evaluated whether kinin receptors are involved in the endothelial vasodilator response to ACh, in which leads to vasorelaxation via NOS activation. As shown in the Figure 2, aortic rings exhibited concentration-dependent vasodilation in response to ACh, which was partially reduced by pre-incubation with the selective inhibitor of nNOS (TRIM; Figures 2A,C) and markedly decreased by the non-selective NOS inhibitor (L-NNA; Figures 2B,D). To assess the contribution of B_1_- and B_2_-kinin receptors in the endothelium-dependent vasodilation response elicited by ACh, aortas were pre-incubated with either a selective B_1_R or B_2_R antagonist. Interestingly, blockage of B_1_R (Figures 2A,B) or B_2_R (Figures 2C, D) led to a significant reduction in ACh-induced vasorelaxation.

To better understand the individual contribution of eNOS and nNOS in the reduced vasorelaxation response to ACh upon B_1_- and B_2_-kinin receptor blockage, we performed experiments combining kinin receptor antagonists and NOS inhibitors. Our results show that pre-incubation with Des-Arg^10^ HOE 140 in combination with L-NNA (Figure 2B) or HOE 140 plus L-NNA (Figure 2D) fully abolished the vasorelaxation induced by ACh. However, pre-incubation with TRIM in combination with kinin receptors antagonists did not potentiate the inhibitory effect of Des-Arg^10^ HOE 140 (Figure 2A) or HOE 140 (Figure 2C) on vasodilator responses.

**Figure 3:**
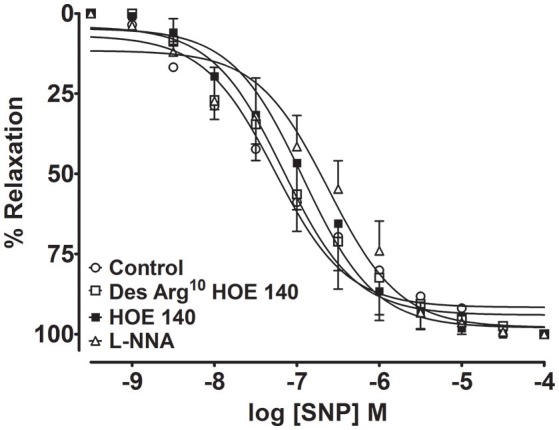
**Endothelium-independent vascular relaxation mediated by NO· donor (sodium nitroprusside, SNP) was not modified in aortic rings treated with B_**1**_- and B_**2**_- kinin receptor antagonists**. Cumulative concentration-response curves for SNP were determined using an antagonist of the B_1_-kinin receptor (Des-Arg^10^ HOE 140; 1 μM), B_2_-kinin receptor (HOE 140; 1 μM) or NOS inhibitor (L-NNA; 1 μM). The results are expressed as mean ± SEM for 4–6 experiments in each group.

To assess whether B_1_- and B_2_-kinin receptors affect endothelium-independent vasorelaxation, the NO· donor SNP was used to induce relaxation in endothelium-denuded aortic rings. As shown in the Figure 3, pre-incubation of B_1_- and B_2_-kinin receptors antagonists did not change the SNP-induced relaxation. As expected, non-selective NOS inhibition did not affect the endothelium-independent relaxation induced by the NO· donor. Collectively, these findings suggest that blockade of kinin receptors affects ACh-induced vascular relaxation via impairment of nNOS activation.

### Genetic deletion of B_1_- or B_2_-kinin receptors impairs ACh-induced vasorelaxation

**Figure 4:**
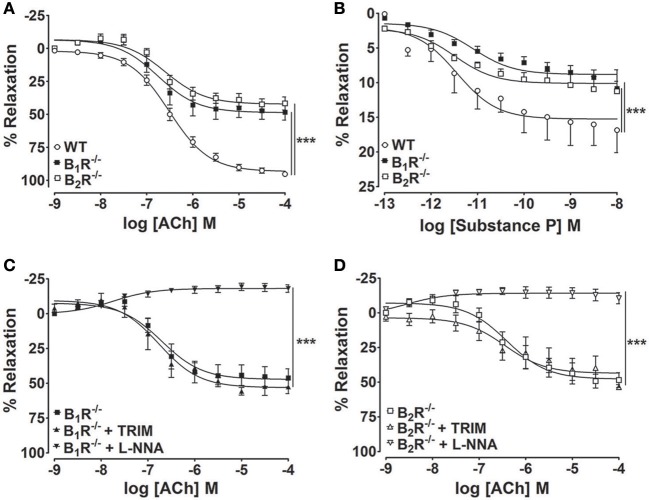
**Genetic deletion of B_**1**_- or B_**2**_-kinin receptors impairs ACh-and Substance P-induced vasodilation**. Cumulative concentration-response curves for Ach **(A)** and substance P **(B)** in B_1_R^−/−^ and B_2_R^−/−^ mice. Further ACh-induced vasodilation was determined in the presence and absence of selective inhibitors of nNOS (**C,D**; TRIM; 100 μM) or constitutive NOS (**C,D**; L-NNA; 1 μM). The results are expressed as mean ± SEM for 8–10 experiments in each group. ^***^P < 0.001.

**Figure 5:**
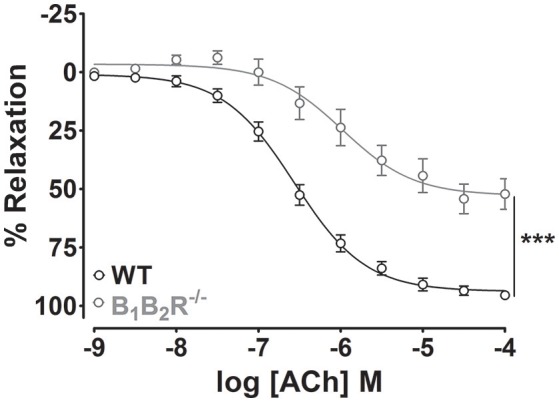
**Genetic deletion of B_**1**_- and B_**2**_-kinin receptors (B_**1**_B_**2**_R^**−/−**^) does not amplify the impairment of ACh-induced vasodilation**. Cumulative concentration-response curves for ACh in WT (*n* = 15) and B_1_B_2_R^−/−^ (*n* = 11) mice. ^***^P < 0.001.

To unequivocally demonstrate that kinin receptors contribute to the endothelium-dependent vasodilation elicited by ACh, we made use of knock-out mice that lack either B_1_- or B_2_-kinin receptors. As shown in Figure 4A, impaired ACh-induced vasorelaxation was found in B_1_R^−/−^ and B_2_R^−/−^ mice compared with WT. Furthermore, substance P, another endothelium-dependent vasodilator via NOS activation, induced a concentration-dependent vasodilation in WT mice, which was significantly decreased in B_1_R^−/−^ and B_2_R^−/−^ (Figure 4B). Consistent with pharmacological approach, the selective inhibition of nNOS did not potentiate the impaired ACh-induced vasodilation in B_1_R^−/−^ or B_2_R^−/−^ mice whereas pre-incubation with L-NNA fully abolished the residual ACh-induced vasodilation (Figures 4C,D). Moreover, impaired ACh-induced vasorelaxation was not amplified in B_1_B_2_R^−/−^ (E_max_: 52.1 ± 6.5%, Figure 5) when compared with B_1_R^−/−^ or B_2_R^−/−^ (Table 1). Overall, these data validate our findings with pharmacological approach and indicate that uncoupled nNOS activity is the main cause of endothelial dysfunction in aorta of B_1_R^−/−^ and B_2_R^−/−^ mice.

**Table 1:** **E_**max**_ values of ACh-induced relaxation in all experimental conditions**.

**Groups**	**E_max_ (%)**	**Groups**	**E_max_ (%)**
Control	96.6 ± 1.1	WT	95.5 ± 1.3
TRIM	47.9 ± 6.3		
L-NNA	14.3 ± 9.5		
Des-Arg^10^ HOE 140	56.4 ± 8.3	B_1_R^−/−^	47.3 ± 6.3
HOE 140	62.2 ± 3.3	B_2_R^−/−^	45.1 ± 5.2
Des-Arg^10^ HOE 140 + TRIM	40.1 ± 7.3	B_1_R^−/−^ + TRIM	52.6 ± 4.8
Des-Arg^10^ HOE 140 + L-NNA	−25.9 ± 4.6	B_1_R^−/−^ + L-NNA	−18.0 ± 2.7
HOE 140 + TRIM	54.8 ± 6.4	B_2_R^−/−^ + TRIM	53.5 ± 1.8
HOE 140 + L-NNA	−22.8 ± 12.0	B_2_R^−/−^ + L-NNA	−10.5 ± 4

*The results are expressed as mean ± SEM*.

### Contribution of B_1_- or B_2_-kinin receptors on NO· production and superoxide generation

**Figure 6:**
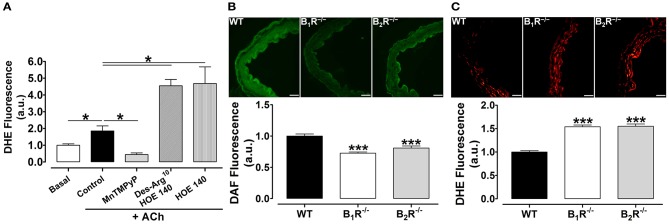
**Contribution of B_**1**_ - or B_**2**_-kinin receptors on NO production and ROS generation. (A)** DHE fluorescence in aortic rings from WT mice incubated in presence or absence of Des-Arg^10^ HOE 140, HOE 140 (1 μM) or MnTMPyP (10 μM) for 30 min prior to ACh stimulation (10 μM, 30 min). **(B,C, Top)** Representative images of aortic rings from WT, B_1_R^−/−^ and B_2_R^−/−^ mice loaded with NO· indicator **(B)** and superoxide indicator **(C)**. **(B,C, Bottom)** Quantitative analysis of DAF **(B)** and DHE fluorescence **(C)**. *n* = 15–20 aortic rings analyzed from 3 animals. Scale bar = 20 μm. The results are expressed as mean ± SEM. ^*^P < 0.05; ^***^P < 0.001 vs. WT.

Taking into account that oxidative stress is a major contributor and marker of endothelial dysfunction, we next investigated the superoxide generation in thoracic aortic rings. As shown in the Figure 6A, ACh was able to raise superoxide levels, in which were fully restored by the SOD-like membrane permeable compound, MnTMPyP, a superoxide scavenger. In line with reduced vasodilation, acute pharmacological blockage of B_1_- or B_2_-kinin receptors markedly potentiates DHE fluorescence when compared to ACh-stimulated aortic rings in the absence of kinin receptors antagonists (Figure 6A). Moreover, we also show that NO· levels were significantly decreased in rings from B_1_R^−/−^ and B_2_R^−/−^ mice compared to WT (Figure 6B). Accordingly, B_1_R^−/−^ and B_2_R^−/−^ aortas exhibited an increased superoxide levels when compared to WT (Figure 6C). Altogether, our data show that reduced NO· bioavailability and greater ROS generation are linked with the impaired endothelium-dependent vasorelaxation of B_1_R^−/−^ and B_2_R^−/−^ mice.

### SOD mimetic MnTMPyP rescues impaired endothelium-dependent vasorelaxation in mice lacking B_1_- or B_2_-kinin receptors

**Figure 7:**
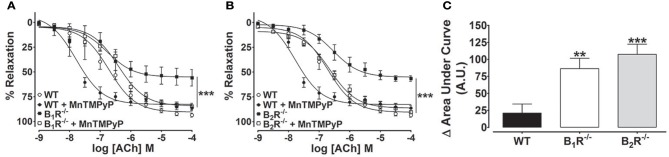
**Superoxide dismutase mimetic (MnTMPyP) rescues impaired ACh-induced vasodilation in mice lacking B_**1**_- or B_**2**_-kinin receptors**. Cumulative concentration-response curves for ACh in B_1_R^−/−^ **(A)** and B_2_R^−/−^ mice **(B)** in the presence and absence of MnTMPyP (10 μM). Delta of the area under the curve representing the enhancement MntMPyP effect on the vasodilation of ACh in WT, B_1_R^−/−^ and B_2_R^−/−^mice **(C)**. The results are expressed as mean ± SEM for 5–10 experiments in each group. ^**^P < 0.01 and ^***^P < 0.001 vs. WT.

In order to confirm whether oxidative stress is involved in the endothelial dysfunction of B_1_R^−/−^ and B_2_R^−/−^ mice, ACh-induced vasorelaxation curves were performed in the presence of MnTMPyP. As shown in the Figures 7A,B, ACh-induced vasorelaxation was leftward shifted in MnTMPyP-treated aortic rings when compared to non-treated vessels of WT mice. Interestingly, in aortic rings from B_1_R^−/−^ and B_2_R^−/−^ mice, MnTMPyP restored the ACh-induced vasorelaxation (Figures 7A,B). The difference of the area under curve shows that the SOD mimetic enhanced ACh-induced vasorelaxation in all tested groups; however, B_1_R^−/−^ and B_2_R^−/−^ mice reached a significant enhancement when compared to WT mice (Figure 7C). Taken together, these data support the oxidative pathogenesis hypothesis of the endothelial dysfunction found in B_1_R^−/−^ and B_2_R^−/−^ mice.

## Discussion

The main findings of this study are as follows: (1) both B_1_- and B_2_-kinin receptors form heteromers with nNOS and eNOS in thoracic aorta; (2) B_1_- and B_2_-kinin receptors equally play an important role in the regulation of endothelium-dependent vasodilation of ACh through nNOS activity; (3) the oxidative stress is closely related with the onset of endothelial dysfunction in aorta of B_1_R^−/−^ and B_2_R^−/−^ mice.

Kinin receptors have been reported to play a pivotal role in numerous vascular responses (Berthiaume et al., 1997; Felipe et al., 2007). Although it remains controversial whether B_1_R^−/−^ and B_2_R^−/−^ mice are normotensive (Milia et al., 2001; Trabold et al., 2002; Abadir et al., 2003) or hypertensive (Madeddu et al., 1997, 1998; Emanueli et al., 1998, 1999), these mice exhibit increased susceptibility to develop hypertension in response to different stressor stimuli (Alfie et al., 1997; Madeddu et al., 1997; Duka et al., 2001). Furthermore, it was previously demonstrated that mice lacking B_1_- or B_2_-kinin receptors display a decreased NO· bioavailability, despite the increased enzymatic activity of the constitutive NOS isoform (Loiola et al., 2011). Thus, these findings indicate that the vascular NOS activity might be uncoupled in B_1_R^−/−^ and B_2_R^−/−^ mice. Moreover, whether ROS generation is involved, or not, in this vascular dysfunction remains unknown. Besides of these important issues, identification of the uncoupled NOS isoform represents another relevant question that could explain the underlying mechanisms and molecular basis of NOS uncoupling in B_1_R^−/−^ and B_2_R^−/−^ mice.

Ca^2+^-dependent NOS, which include eNOS and nNOS, are tightly regulated by several regulatory sites. Furthermore, new molecular regulators have been identified affecting their activities and, consequently, the amount of critical end-products for vascular function. Notably, protein-protein interactions have been proposed as an important mechanism that regulates the activity of NOS (Kone et al., 2003). Indeed, several studies have already described the functional relevance of physical interactions between NOS with a wide variety of structural and regulatory proteins such as, calmodulin (Rizzo et al., 1998), caveolin-1 (Ghosh et al., 1998), heat shock protein 90 (Russell et al., 2000), α1A adrenergic receptors (Pupo and Minneman, 2002), and angiotensin II receptor type 1 (AbdAlla et al., 2000). Although the heteromerization of B_2_-kinin receptor with nNOS and eNOS was previously reported (Ju et al., 1998; Golser et al., 2000), this study is the first to demonstrate B_1_R–eNOS/nNOS heteromerization. Moreover, although we are the first group to actually demonstrate such macromolecular protein complexes in native vascular tissue, if these interactions are functionally coupled to each other and, more importantly, whether this affects vascular function, it has remained unknown until now.

Here, we showed that eNOS and nNOS inhibition strongly decreases the ACh-induced vasorelaxation, confirming the large contribution of both constitutive NOS as the main source of NO· production. Intriguingly, pharmacologic antagonism or genetic ablation of B_1_- or B_2_-kinin receptors decreased the endothelium-dependent vasorelaxation response to ACh. Therefore, our results show that kinin receptors act as regulators of NOS activity in endothelial cells. To identify whether kinin receptors differently modulate eNOS and nNOS activities, the vasorelaxation response to ACh was assessed combining kinin receptors antagonists and NOS inhibitors. Our results demonstrated that B_1_- and B_2_-kinin receptors blockage fully abolished the relaxation upon non-selective NOS inhibition whereas nNOS inhibition did not potentiate the reduction of vasorelaxation induced by kinin receptors antagonism. In addition, we found similar pattern of vascular response in aorta from B_1_R^−/−^ and B_2_R^−/−^ mice. It is worth noting that non-selective NOS inhibition and B_1_- or B_2_-kinin receptors antagonists led to vascular contraction. These vascular responses further support that inhibition of the main vasodilation pathway favors contractile mechanisms, in which the oxidative stress is well-known to play a dysfunctional role in several vascular disorders (Johansson et al., 2005; Loria et al., 2014). Moreover, a reasonable explanation for selecting ACh as the agonist to trigger the vasorelaxation in the present study is the well-known NOS activation by muscarinic pathway whereas, the vascular response of agonists of kinin receptors is still controversial in aorta of different species, where it produces vasoconstriction (Levesque et al., 1993; Felipe et al., 2007) or vasodilation (Tsutsui et al., 2000). Furthermore, previous evidence has already demonstrated the absence of protein-protein interactions between kinin and muscarinic receptors (Willars et al., 1999). Importantly, we also validated the vascular dysfunction in B_1_R^−/−^ and B_2_R^−/−^ mice assessing the vasodilation induced by another well-known NOS activator, substance P. Taken together, our results show that B_1_- and B_2_-kinin receptors represent a functional regulatory domain of NOS activity. Moreover, our findings indicate that eNOS activity remained unaffected whereas the loss of nNOS function may represent a potential uncoupled activity.

Taking into account that B_1_R and B_2_R receptors are expressed in endothelial and smooth muscle layers (Leeb-Lundberg et al., 2005), kinin receptors may also affect the vascular relaxation by changing the responsiveness to NO· or, by acting on other targets resident in the vascular smooth muscle. To test this hypothesis, the vasodilator response to an exogenous NO· donor was used. Our results showed that kinin receptors did not affect the vasodilator response to NO· donor, which indicate a preserved smooth muscle function. Therefore, our findings strongly indicate that the endothelial dysfunction is the main cause of impaired vascular relaxation in aorta of B_1_R^−/−^ and B_2_R^−/−^ mice. Importantly, a cross-talk between B_1_R and B_2_R has been reported (Barki-Harrington et al., 2003), indicating an interdependence with each other for the proliferative response. Moreover, it has been also shown that B_1_B_2_R^−/−^ mice display protection against endotoxin-induced hypotension (Cayla et al., 2007) and obesity induced by high fat diet (Morais et al., 2015), while there are evidence showing amplified renal injury after ischemia/reperfusion (Kakoki et al., 2007) and increased nephropathy, neuropathy and bone mineral loss in Akita diabetic mice (Kakoki et al., 2010). Thus, in the present study we show that B_1_B_2_R^−/−^ mice present similar level of vascular dysfunction as found in mice lacking B_1_R or B_2_R, and therefore ruling out a possible synergistic response between these receptors in this mechanism. Although still under debate whether endothelial dysfunction is a primary cause or consequence of vascular diseases, such vascular dysfunction is generally associated with decreased NO· bioavailability and increased superoxide generation which ultimately lead to an impaired endothelium-dependent vasorelaxation (Vanhoutte et al., 2017).

Under physiological conditions, the activity of eNOS is able to produce NO·, whereas nNOS activity produces NO· and hydrogen peroxide (H_2_O_2_; Rosen et al., 2002; Gao et al., 2007). However, during certain pathological stimuli, the activity of eNOS and nNOS is drastically modified and becomes primarily uncoupled, producing a large amount of ROS, mainly, superoxide (Vasquez-Vivar et al., 1998; Weaver et al., 2005). Consistent with impaired vasodilation, acute blockage of kinin receptors markedly potentiate ACh-induced superoxide generation in aorta of WT mice. Additionally, we also demonstrated that B_1_R^−/−^ and B_2_R^−/−^ mice show a reduced NO· bioavailability associated with marked increase in intracellular levels of superoxide. Therefore, although the enhanced constitutive NOS activity in vascular tissue of B_1_R^−/−^ and B_2_R^−/−^ mice, our findings indicate that the uncoupled activity of nNOS represents a remarkable source of superoxide and, may be the major cause of the endothelial dysfunction in B_1_R^−/−^ and B_2_R^−/−^ mice.

Indeed, the oxidative stress is the major cause of endothelial dysfunction in several forms of vascular diseases, mainly via disruption of NO· signaling pathway (Drummond et al., 2011; Silva et al., 2016). Therefore, enhanced superoxide anion dismutation through the SOD mimetic can prevents the inactivation of NO· and formation of the highly reactive intermediate peroxynitrite (Weaver et al., 2005), and thus yielding augmented H_2_O_2_ concentration, an endothelium-dependent relaxing factor in aorta (Capettini et al., 2008, 2010). Accordingly, the left-shifted ACh-induced vasodilation in WT may be explained by the above factors. Moreover, the stronger enhancement of vascular relaxation induced by the SOD mimetic in B_1_R^−/−^ and B_2_R^−/−^ mice than in WT mice further support our hypothesis of oxidative stress involvement in the endothelial dysfunction. These findings are consistent with previous studies, in which the exogenous SOD mimetic restores endothelium-dependent vasodilation due increased NO· bioavailability (Fontana et al., 1999; Braga et al., 2015).

nNOS uncoupling has been implicated in several endothelium-dependent vascular disorders such as, in mesenteric artery of DOCA-salt hypertensive mice (Silva et al., 2016), thoracic aorta subjected to experimental atherosclerosis model (Capettini et al., 2011) and penile arteries of obese Zucker rat (Sánchez et al., 2012). Accordingly, deficient mice of B_1_-kinin receptor has been described to aggravate atherosclerosis and the development of abdominal aorta aneurysms in ApoE^−/−^ mice under cholesterol rich-diet (Merino et al., 2009). B_2_-kinin receptor deficient mice also demonstrate higher blood pressure in response to chronic excess of angiotensin II or dietary salt (Madeddu et al., 1997) and chronic mineralocorticoid excess (Emanueli et al., 1998). Moreover, pharmacologic blockade or genetic disruption of the B_2_-kinin receptor accelerates the development of renovascular hypertension induced by clipping of the left renal artery (Madeddu et al., 1998). On the other hand, a recently study demonstrated that B_2_-kinin receptor antagonist inhibited AngII-induced neutrophil activation and inflammatory phenotype in ApoE^−/−^ mice, thus suggesting B_2_-kinin receptor antagonism as potential therapy for abdominal aortic aneurysm (Moran et al., 2016). Therefore, further studies are necessary in disease models to test the hypothesis whether the conformational rearrangement of protein-protein interactions, involving kinin receptors and NOS isoforms, is relevant to vascular dysfunction.

In summary, we provide data supporting a novel implication of kinin receptors in the endothelial dysfunction. We report here that B_1_- and B_2_-kinin receptors regulate the endothelium-dependent vasodilation of ACh through nNOS activity and indicate that molecular disturbance of short-range interactions between B_1_- and B_2_-kinin receptors with nNOS is involved in the oxidative pathogenesis of endothelial dysfunction.

## Author contributions

TM participated in all steps of this study. GC and IJ performed experiments. SL, LC, VL, JC, EC, JLP, and JBP contributed to the experimental design, data analyses, data interpretation, and the preparation and revision of the manuscript.

## Funding

This study was supported by Conselho Nacional de Desenvolvimento Científico e Tecnológico (CNPq, Brazil), PROMOB–Coordenação de Aperfeiçoamento de Pessoal de Nível Superior e Fundação de Apoio à Equipe e à Inovação Tecnológica do Estado de Sergipe (CAPES/FAPITEC/SE, Brazil), Fundação de Amparo à Pesquisa do estado de Minas Gerais (FAPEMIG/MG, Brazil). JC and EC are CNPq research fellow.

### Conflict of interest statement

The authors declare that the research was conducted in the absence of any commercial or financial relationships that could be construed as a potential conflict of interest.
